# Characteristics Regarding Lift-Off Intersection of Pulse-Modulation Eddy Current Signals for Evaluation of Hidden Thickness Loss in Cladded Conductors

**DOI:** 10.3390/s19194102

**Published:** 2019-09-23

**Authors:** Yong Li, Yi Wang, Zhengshuai Liu, Ilham Mukriz Zainal Abidin, Zhenmao Chen

**Affiliations:** 1 State Key Laboratory for Strength and Vibration of Mechanical Structures, Shaanxi Engineering Research Centre of NDT and Structural Integrity Evaluation, School of Aerospace Engineering, Xi’an Jiaotong University, Xi’an 710049, China; wybit2008@stu.xjtu.edu.cn (Y.W.); liuzhengshuai@stu.xjtu.edu.cn (Z.L.); chenzm@mail.xjtu.edu.cn (Z.C.); 2 Leading Edge NDT Technology (LENDT) Group, Malaysian Nuclear Agency, Bangi 43000, Kajang, Selangor, Malaysia; mukriz@nuclearmalaysia.gov.my

**Keywords:** pulse-modulation eddy current inspection, lift-off intersection, cladded conductor, thickness loss, analytical modeling

## Abstract

The cladded conductor is broadly utilized in engineering fields, such as aerospace, energy, and petrochemical; however, it is vulnerable to thickness loss occurring in the clad layer and nonconductive protection coating due to abrasive and corrosive environments. Such a flaw severely undermines the integrity and safety of the mechanical structures. Therefore, evaluating the thickness loss hidden inside cladded conductors via reliable nondestructive evaluation techniques is imperative. This paper intensively investigates the pulse-modulation eddy current technique (PMEC) for the assessment of thickness loss in a cladded conductor. An analytical model of the ferrite-cored probe is established for analyzing PMEC signals and characteristics of lift-off intersection (LOI) in testing signals. Experiments are conducted for evaluation of the thickness loss in cladded conductors. An inverse scheme based on LOI for estimation of the thickness-loss depth is proposed and further verified. Through simulations and experiments, it is found that the influences of the thickness loss in the clad layer and protective coating on the PMEC signals can be decoupled in virtue of the LOI characteristics. Based on LOI, the hidden thickness loss can be efficiently evaluated without much of a reduction in accuracy by using the PMEC probe for dedicated inspection of the cladded conductor.

## 1. Introduction

In an effort to enhance resistance to corrosion and abrasion, the critical conductive components of nonferromagnetic materials in engineering structures employed in fields such as energy, aerospace, and petrochemical, are cladded with a layer of distinct/premium nonferromagnetic materials, including the copper alloy [1,2]. Furthermore, a nonconductive protection coating is usually deployed over the clad layer for further protection. This makes the cladded conductor regarded as a stratified structural system consisting of the protective coating (upper layer, nonconductive), clad layer (middle layer, conductive), and substrate (bottom layer, conductive). 

During fabrication (using the techniques of diffusion, explosion-bonding and lasers, etc.) and practical service, thickness loss is usually found to occur in the surfaces of the protective coating and clad layer of the cladded conductor, which severely influences the structural integrity and ultimately undermines the mechanical strength and safety of the mechanical structures [3]. A featured example is a planar cladded conductor employed in aerospace engineering structures such as the unmanned aerial vehicle (UAV), which consists of a substrate of aluminum alloy, clad layer of copper alloy, and nonconductive protection coating over the clad layer. The thickness loss in the surface of the protective coating and clad layer leaves the cladded conductor as well as the UAV vulnerable to structural failure and catastrophic accidents. Therefore, it is indispensable to periodically inspect and quantitatively evaluate the thickness loss hidden inside the cladded conductor via the effective non-destructive evaluation (NDE) techniques, which benefit structural monitoring in terms of the integrity as well as the mechanical strength of the featured cladded conductor. Whereas, the structural characteristics of the cladded conductor make NDE techniques, such as ultrasonic testing (UT) [4], which is normally adopted for clad layer thickness checking after fabrication of the clad layer, inapplicable for simultaneous evaluation of thickness loss of the protective coating and clad layer. In light of this, electromagnetic NDE methods involving eddy current testing (EC) [5,6] and pulsed eddy current testing (PEC) [7,8,9], which have been found to be capable of detecting and evaluating surface and subsurface defects in the conductive structures, could be promising and preferable for noninvasive interrogation of the thickness loss in cladded conductors. In order to further enhance the inspection sensitivity and evaluation accuracy of the two methods, the pulse-modulation eddy current technique (PMEC) [10] has been proposed. It pushes the boundary of PEC and has been identified to be advantageous to the aforementioned methods, particularly in terms of dedicated inspection, assessment, and imaging of defects in conductors [11].

It is noteworthy that the thickness loss in cladded conductors essentially leads to the decrease in thickness of the protective coating and clad layer from their surfaces, giving rise to variation in the probe lift-off (i.e., the distance between the probe bottom and the surface of the clad layer). Therefore, such a flaw is regarded as the composite defect, which involves the lift-off variation and metal loss, and the influences of the thickness-loss depths on the testing signal are coupled. This leaves the traditional signal processing techniques of PMEC for evaluation of defects in conductors vulnerable to significant reductions in accuracy regarding assessment of defect parameters due to the so-called lift-off noise [12,13,14]. In an effort to mitigate the lift-off noise previously found in EC and PEC, lift-off intersection (LOI) has been exploited [15]. It is a physical phenomenon revealing that when the probe lift-off varies during inspection, the testing signals for different lift-off cases intersect at a train of points whose magnitudes and time instants are immune to the probe lift-off but are dependent on properties of the conductor under inspection [16]. Previous research has intensively investigated the LOI of EC and PEC. Following the theoretical and experimental analysis of characteristics of LOI in EC signals by Mandache et al. [17], Tian and Li et al. investigated the time instant of the LOI point in PEC signals and its correlation with probe lift-off and metal conductivity [18]. Fan et al. scrutinized the LOI of PEC for the measurement of conductor thickness through analytical modeling and experiments [19,20,21]. Li et al. proposed an inverse scheme in conjunction with both the magnitude and time instant of the LOI point for evaluation of coated conductors via gradient-field PEC [22]. However, to the authors’ knowledge, few studies have been carried out in a bid to: (1) investigate characteristics of LOI in PMEC signals, or (2) propose an efficient evaluation method for simultaneous assessment of the thickness loss in the clad layer and protective coating of the cladded conductor based on the properties of LOI points in PMEC signals. 

In light of this, in this paper the characteristics of LOI in PMEC signals for quantification of the thickness loss in the cladded conductor, whose structure includes a nonconductive protection coating, nonferromagnetic clad layer, and substrate were intensively explored. An analytical model concerning a ferrite-cored PMEC probe over a cladded conductor was established based on the extended truncated region eigenfunction expansion (ETREE) [23]. Following this, the characteristics of PMEC responses to the featured cladded conductor subject to the thickness loss and LOI of PMEC were analyzed via theoretical simulations. In parallel, experiments were conducted for investigation regarding PMEC evaluation of the thickness loss in the featured cladded conductor. The feasibility of the PMEC probe together with the LOI-based inversion for simultaneous evaluation of the thickness loss in the clad layer and protective coating was further identified. The rest of the paper is organized as follows: Section 2 elaborates the formulation of closed-form expressions of PMEC responses from a ferrite-cored probe to the cladded conductor. The investigation of features of the testing signals and LOI of PMEC is presented in Section 3. It is followed by an experimental study concerning quantitative evaluation of the thickness loss in the featured cladded conductor via PMEC, which is presented in Section 4.

## 2. Field Formulation

**Figure 1:**
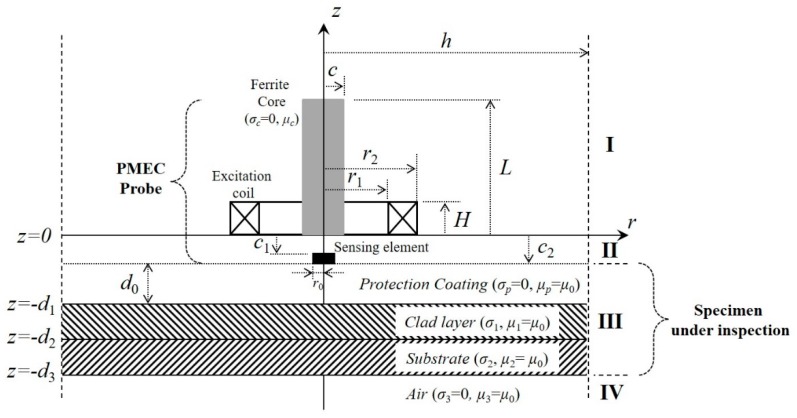
The 2D axisymmetric model of a ferrite-cored pulse-modulation eddy current technique (PMEC) probe placed over the protective coating of a cladded conductor.

Suppose that a ferrite-cored PMEC probe is deployed over the protective coating of a cladded conductor, which is portrayed in Figure 1. The probe comprises: (1) a ferrite-cored excitation coil for generation of the incident magnetic field; and (2) a solid-state magnetic field sensor, which is placed at the bottom center of the ferrite core and used for sensing the net magnetic field (superposition of the incident and eddy-current-induced magnetic fields). It is assumed that the length and width of the thickness loss are considerably larger than the outer diameter of the excitation coil. Even though with regard to ferrite-cored coils, the closed-form expression of the net magnetic field in the solution region can be formulated as per Reference [24] for traditional EC, the field formulation is further extended to PMEC via ETREE modeling [23].

Based on ETREE modeling for transient eddy current inspection [11], the closed-form expression of *z*-component of the net magnetic field at an arbitrary position in Region II can be written as:

(1) $$B_{z}\left(r,z,t\right)=4\mu_{0}\tau I\left(t\right)⊗\Lambda \left(r,z,t\right)=4\mu_{0}\tau I\left(t\right)⊗IFT\left[\Lambda \left(r,z,\omega \right)\right]$$

where ⊗ denotes circular convolution; *μ*_0_ is the vacuum permeability; *I*(*t*) stands for the PMEC excitation current signal whose expression can be found in Reference [10]; *τ* is the density of the coil winding, *τ* = *N*[*H*(*r*_2_ − *r*_1_)]^−1^, where *N* is the number of turns of the excitation coil; and Λ(*r*, *z*, *t*) is the function depicting the field response to the conductor when the ferrite-cored excitation coil is driven by the impulse current in the Dirac delta function of time. This can be readily computed with its spectral form Λ(*r*, *z*, *ω*) in conjunction with the Inverse Fourier Transform (IFT) [25]. Note that in Equation (1), *ω* denotes the angular frequency of each harmonic within the PMEC excitation current. Based on References [24], [26], and [27], Λ(*r*, *z*, *ω*) is formulated in matrix notation as:

(2) $$\Lambda \left(r,z,\omega \right)=J_{0}\left(\kappa r\right)\left[e^{\kappa z}+e^{-\kappa z}\Gamma \right]E\cdot C$$

where *J_m_* is the Bessel function of the first kind; **κ** is the row vector with the element of *κ_i_*, *i* = 1, 2, 3 … *N_s_* (the number of elements); *κ_i_* is the positive root of *J*_1_(*κ_i_h*) = 0; the superscript **T** denotes transpose; $e^{\kappa z}$ and $e^{-\kappa z}$ are *N_s_* × *N_s_* diagonal matrices with the diagonal elements written as $e^{\kappa_{i}z}$ and $e^{-\kappa_{i}z}$, respectively; **E** is the *N_s_* × *N_s_* diagonal matrix whose diagonal element is computed by: $E_{i}={\left[h^{2}J_{0}^{2}\left(\kappa_{i}h\right)\right]}^{-1}$; and **Γ** is the matrix of the conductor reflection coefficient which is formulated as:

(3) $$\left\{ \begin{array}{l} \Gamma =\left[M_{0}e^{-\left(\kappa +\lambda_{1}\right)d_{1}}U+N_{0}e^{-\left(\kappa -\lambda_{1}\right)d_{1}}V\right]{\left[N_{0}e^{\left(\kappa -\lambda_{1}\right)d_{1}}U+M_{0}e^{\left(\kappa +\lambda_{1}\right)d_{1}}V\right]}^{-1}\\ M_{0}=\kappa \lambda_{1}^{-1}-\mu_{1}^{-1}I\;\;N_{0}=\kappa \lambda_{1}^{-1}+\mu_{1}^{-1}I \end{array}\right.$$

where **I** denotes the identity matrix. The matrices **U** and **V** are expressed as:

(4) $$\left\{ \begin{array}{l} U=\left[N_{1}N_{2}e^{\left(\lambda_{1}-\lambda_{2}\right)d_{2}+\left(\lambda_{2}-\lambda_{3}\right)d_{3}}+M_{1}M_{2}e^{\left(\lambda_{1}+\lambda_{2}\right)d_{2}-\left(\lambda_{2}+\lambda_{3}\right)d_{3}}\right]/4\\ V=\left[M_{1}N_{2}e^{-\left(\lambda_{1}+\lambda_{2}\right)d_{2}+\left(\lambda_{2}-\lambda_{3}\right)d_{3}}+N_{1}M_{2}e^{\left(\lambda_{2}-\lambda_{1}\right)d_{2}-\left(\lambda_{2}+\lambda_{3}\right)d_{3}}\right]/4\\ M_{1}=\lambda_{1}\lambda_{2}^{-1}-\mu_{1}\mu_{2}^{-1}I\;\;N_{1}=\lambda_{1}\lambda_{2}^{-1}+\mu_{1}\mu_{2}^{-1}I\\ M_{2}=\lambda_{2}\lambda_{3}^{-1}-\mu_{2}\mu_{3}^{-1}I\;\;N_{2}=\lambda_{2}\lambda_{3}^{-1}+\mu_{2}\mu_{3}^{-1}I \end{array}\right.$$

Note that in Equations (3) and (4) all matrices are *N_s_* × *N_s_* diagonal matrices. The diagonal element in **λ_n_** (n = 1, 2, 3) is computed via: $\lambda_{ni}=\sqrt{\kappa_{i}^{2}+j\omega \mu_{0}\mu_{n}\sigma_{n}}$.

The other matrix in Equation (2) includes **C** which is formulated as:

(5) $$\left\{ \begin{array}{l} C={\left[\alpha e^{qL}\left(a+b\Gamma \right)-\beta e^{-qL}\left(b+a\Gamma \right)\right]}^{-1}\left\{\beta \left[e^{q\left(H-L\right)}-e^{-qL}\right]-\alpha \left[e^{-q\left(H-L\right)}-e^{qL}\right]\right\}q^{-3}D\Omega \\ \alpha =S+T\;\;\;\;\beta =S-T\\ a=T^{-1}+S^{-1}\;\;b=T^{-1}-S^{-1} \end{array}\right.$$

where **q** is the *N_s_* × *N_s_* diagonal matrix. Its diagonal element *q_i_* can be derived by finding the real positive root of the equation:

(6) $$\left\{ \begin{array}{l} \left[J_{1}\left(x\right)Y_{0}\left(x\right)-\mu_{c}^{-1}J_{0}\left(x\right)Y_{1}\left(x\right)\right]J_{1}\left(hc^{-1}x\right)=\left(1-\mu_{c}^{-1}\right)J_{0}\left(x\right)J_{1}\left(x\right)Y_{1}\left(hc^{-1}x\right)\\ q_{i}=xc^{-1} \end{array}\right.$$

where *Y_m_* denotes the Bessel function of the second kind. **D** is the *N_s_* × *N_s_* diagonal matrix with the diagonal element written as:

(7) $$D_{i}={\left\{h^{2}R_{0}^{2}\left(q_{i}h\right)+c^{2}\left(1-\mu_{c}^{-1}\right)\left[\mu_{c}^{-1}J_{0}^{2}\left(q_{i}c\right)-J_{1}^{2}\left(q_{i}c\right)\right]\right\}}^{-1}$$

where

(8) $$R_{0}\left(q_{i}r\right)=\frac{\pi cq_{i}}{2}\left\{\left[\begin{array}{l} J_{1}\left(q_{i}c\right)Y_{0}\left(q_{i}c\right)\\ -\mu_{c}^{-1}J_{0}\left(q_{i}c\right)Y_{1}\left(q_{i}c\right) \end{array}\right]J_{0}\left(q_{i}r\right)+\left(\mu_{c}^{-1}-1\right)J_{0}\left(q_{i}c\right)J_{1}\left(q_{i}c\right)Y_{0}\left(q_{i}r\right)\right\}$$

**S** and **T** are *N_s_* × *N_s_* full matrices whose elements are defined as:

(9) $$S_{ij}=\left\{ \begin{array}{ll} c\kappa_{i}J_{0}\left(\kappa_{i}c\right)J_{1}\left(q_{j}c\right)\left(1-\mu_{c}^{-1}\right)/\left(\kappa_{i}^{2}-q_{j}^{2}\right) & \kappa_{i}\neq q_{j}\\ \left\{h^{2}J_{0}\left(q_{j}h\right)R_{0}(q_{j}h)-c\left[cJ_{1}(q_{j}c)+q_{j}^{-1}J_{0}\left(q_{j}c\right)\right]J_{1}(q_{j}c)\left(1-\mu_{c}^{-1}\right)\right\}/2 & \kappa_{i}=q_{j} \end{array}\right.$$

(10) $$T_{ij}=\left\{ \begin{array}{ll} c\kappa_{i}J_{1}\left(\kappa_{i}c\right)J_{0}\left(q_{j}c\right)\left(1-\mu_{c}^{-1}\right)/\left(\kappa_{i}^{2}-q_{j}^{2}\right) & \kappa_{i}\neq q_{j}\\ \left[h^{2}J_{0}\left(q_{j}h\right)R_{0}\left(q_{j}h\right)+c^{2}J_{0}^{2}\left(q_{j}c\right)\left(1-\mu_{c}^{-1}\right)\right]/2 & \kappa_{i}=q_{j} \end{array}\right.$$

**Ω** is the *N_s_* × 1 column vector and its element is formulated as:

(11) $$\Omega_{i}=\int_{q_{i}r_{1}}^{q_{i}r_{2}}xR_{1}\left(x\right)dx={\frac{\pi }{2}x\left[H_{0}\left(x\right)R_{1}\left(x\right)-H_{1}\left(x\right)R_{0}\left(x\right)\right]|}_{q_{i}r_{1}}^{q_{i}r_{2}}$$

where *H_m_* denotes the Struve function. Similar to Equation (8), *R*_1_(*x*) is written as:

(12) $$R_{1}\left(x\right)=\frac{\pi cq_{i}}{2}\left\{\left[\begin{array}{l} J_{1}\left(q_{i}c\right)Y_{0}\left(q_{i}c\right)\\ -\mu_{c}^{-1}J_{0}\left(q_{i}c\right)Y_{1}\left(q_{i}c\right) \end{array}\right]J_{1}\left(x\right)+\left(\mu_{c}^{-1}-1\right)J_{0}\left(q_{i}c\right)J_{1}\left(q_{i}c\right)Y_{1}\left(x\right)\right\}$$

In consideration of the dimension of the sensing element of the solid-state magnetic field sensor, the closed-form expression of the PMEC signal can be readily formulated by taking the integral of Λ(*r*, *z*, *ω*) in Equation (1) over the element volume, and is thus written as:

(13) $$B_{PMEC}\left(t\right)=\frac{8\mu_{0}\tau I\left(t\right)}{r_{0}\left(c_{2}-c_{1}\right)}⊗IFT\left\{\Psi \left[\left(e^{-\kappa c_{1}}-e^{-\kappa c_{2}}\right)+\left(e^{\kappa c_{2}}-e^{\kappa c_{1}}\right)\Gamma \right]E^{\prime }\cdot C\right\}$$

where **Ψ** and **E′** are the 1 × *N_s_* row vector and *N_s_* × *N_s_* diagonal matrix, respectively. Their elements are defined as:

(14) $$\Psi_{i}=\kappa_{i}^{-1}J_{1}\left(\kappa_{i}r_{0}\right)\;\;E_{i}^{\prime }=\kappa_{i}^{-1}{\left[hJ_{0}\left(\kappa_{i}h\right)\right]}^{-2}$$

$e^{\kappa c_{1}}$, $e^{\kappa c_{2}}$, $e^{-\kappa c_{1}}$, and $e^{-\kappa c_{2}}$ are *N_s_* × *N_s_* diagonal matrices with the diagonal elements written as $e^{\kappa_{i}c_{1}}$, $e^{\kappa_{i}c_{2}}$, $e^{-\kappa_{i}c_{1}}$, and $e^{-\kappa_{i}c_{2}}$, respectively. It is noteworthy that Equation (13) facilitates the prediction of the PMEC response from the probe to a cladded conductor with the thickness loss in the clad layer and protective coating.

## 3. Simulations and Investigation Regarding LOI of PMEC Signals

### 3.1. Simulation Setup and Corroboration

**Figure 2:**
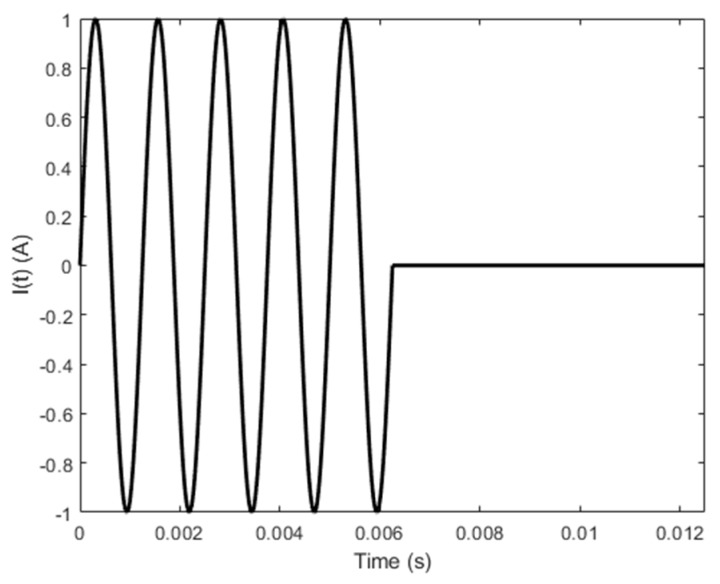
The excitation current driving the ferrite-cored excitation coil of the PMEC probe.

**Figure 3:**
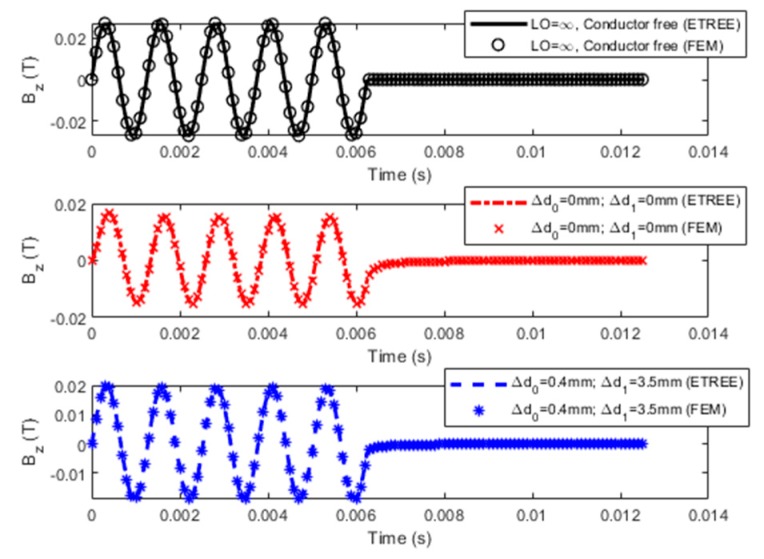
Predicted PMEC signals and comparison with finite element modeling (FEM) results.

A series of simulations based on Equation (13) have been conducted in regard to: (1) PMEC responses to a cladded conductor, where the nonferromagnetic clad layer and nonconductive protection coating are subjected to thickness loss; and (2) LOI characteristics of PMEC. The parameters of the probe and unflawed specimen are tabulated in Table 1 and Table 2, respectively. The current in the pulse modulation waveform for driving the excitation coil is shown in Figure 2. The frequencies of the carrier wave (*f_c_* = 800 Hz) and modulation waves (*f_m_* = 80 Hz) of the current waveform are chosen as per the rule of thumb elaborated in References [10] and [25]. During simulations, in an attempt to simulate the cases with flawed specimens, for the thickness loss in the clad layer, *d*_1_ varies whilst *d*_2_ is fixed. In contrast, *d*_0_ changes with *c*_2_ kept constant to simulate the protection-coating thickness loss. The predicted PMEC signals and their comparison with the results from finite element modeling (FEM) [28] for the same scenario are shown in Figure 3. Note that in Figure 3, LO denotes the probe lift-off. ∆*d*_0_ and ∆*d*_1_ are variations in the protective coating and clad layer, respectively.

It can be observed from Figure 3, that the PMEC signals predicted using Equation (13) formulated via ETREE modeling are in good agreement with those from FEM. The relative error is less than 0.1%. Further comparison is carried out regarding the computation time. It is found that the computation time of ETREE is less than 1 s, whilst it takes more than 800 s for FEM to predict the signal for the same simulation scenario. Complementary to a previous study regarding the air-cored PMEC probe [11], the high efficiency of ETREE in simulations of testing signals is further confirmed for the ferrite-cored probe.

It is also noticeable from Figure 3 that, similar to the air-cored PMEC probe, the amplitude of the PMEC signal from the ferrite-cored probe decreases as the probe is deployed above the cladded conductor due to the repulsive effect from the secondary magnetic field induced by eddy currents in the conductive media. When the thickness of the clad layer drops and the probe lift-off is fixed (constant *d*_0_), the magnitude of the PMEC signal of the net magnetic field rises because the thickness loss brings about a decrease in the density of eddy current and thus a decline in the secondary magnetic field. Further investigation indicates that for the case with the constant *d*_1_, the drop in the probe lift-off gives rise to the decrease in the signal amplitude. This implies that the influence on the PMEC signal from the thickness loss in the clad layer opposes that from the protection-coating thickness loss, which could subsequently lead to difficulty in the simultaneous evaluation of thicknesses of the clad layer and protective coating. This issue could be mitigated by introducing LOI, which essentially decouples the influences from the thickness loss of the clad layer and protective coating.

### 3.2. Characteristics of LOI of PMEC Signals 

**Figure 4:**
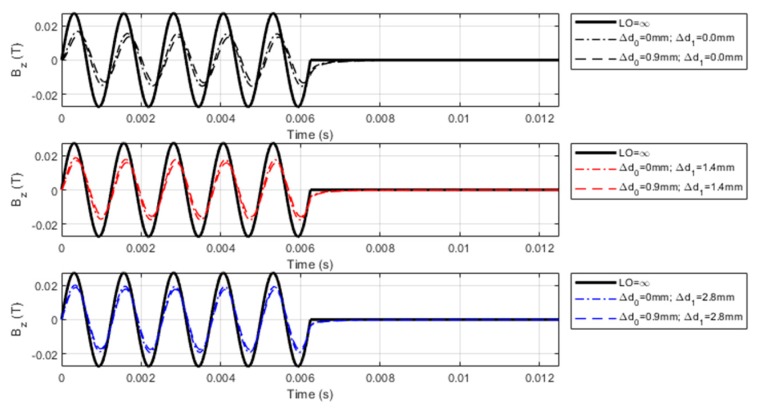
Computed PMEC signals vs. ∆*d*_0_ against different ∆*d*_1._

The thickness loss in the cladded conductor gives rise to the variation in the probe lift-off (LO), which can be expressed as LO = *l*_1_ + *l*_2_ = (*d*_0_ − ∆*d*_0_) + ∆*d*_1_, where *l*_1_ and *l*_2_ depict the contributions of the protective coating and clad layer to LO, respectively. In simulations, ∆*d*_0_ varies from 0 to 0.9 mm, whilst ∆*d*_1_ changes from 0 to 3.5 mm. The computed PMEC responses for different specimen scenarios are exhibited in Figure 4.

It can be observed from Figure 4 that, similar to conventional EC and PEC, LOI occurs in PMEC signals when the probe lift-off varies. Due to the fact that the carrier wave of the PMEC excitation current is sinusoidal, two LOI points can be found within one cycle of the carrier wave. This agrees with the finding regarding LOI in conventional EC. In contrast, compared with PEC, LOI points of PMEC can be readily identified without taking the first-order derivative of the testing signal against time. This benefits the subsequent extraction of the magnitude (M_LOI_) and time instant (T_LOI_) of each LOI point, since the differential processing used in PEC for identification of LOI points is tedious and may aggravate the level of extraneous noise, which already pollutes the PEC signal.

**Figure 5:**
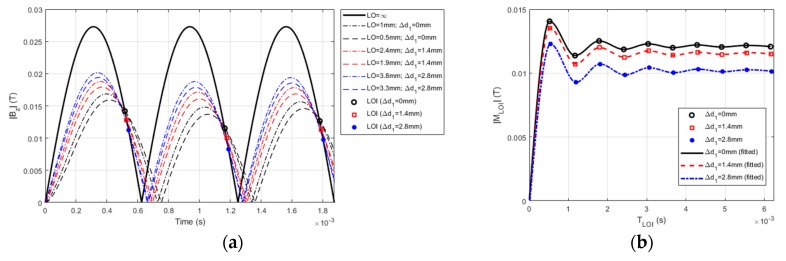
Characteristics of lift-off intersection (LOI) of PMEC signals: (**a**) PMEC signals and LOI points within the observation window (0 ms ≤ *t* ≤ 2 ms); (**b**) M_LOI_ vs. T_LOI_ against ∆*d*_1._

Further investigation has been intensively conducted to analyze the characteristics of LOI points for the clad layer with the thickness loss (CLTL). The PMEC signals (0 ms ≤ *t* ≤ 2 ms) for different ∆*d*_1_ are presented in Figure 5a together with the LOI points. It is noted that in an effort to facilitate the analysis of LOI, every PMEC signal is preprocessed by taking the absolute value of its amplitude, which gives |*B_z_*|. It can be seen from Figure 5a that within the observation window (a quarter of one cycle of the carrier wave) the LOI point varies with ∆*d*_1_, whilst for each CLTL case, M_LOI_ and T_LOI_ are invariant due to the intrinsic characteristics of LOI. This implies the promising application of the LOI point for the evaluation of CLTL and particularly the assessment of ∆*d*_1_. Interestingly, from Figure 5a it can also be observed that for each CLTL case, M_LOI_ varies with T_LOI_. This is distinct from the characteristics of LOI for traditional EC, where with the time-harmonic excitation current, the constant M_LOI_ is found with variable T_LOI_. The reasoning lies in the fact that when the PMEC probe is deployed over a conductor, the transient characteristics of the PMEC signal manifest, particularly at the early stage of the excitation, due to transient electromagnetic induction. This subsequently results in the transient feature of LOI during early cycles of the carrier wave. In a bid to scrutinize the transient characteristics of LOI in PMEC signals, all LOI points within the pulse width (0 ms ≤ *t* ≤ 6.3 ms) of the PMEC excitation current have been extracted. These points with different M_LOI_ and T_LOI_ are portrayed in Figure 5b, along with the fitted curve for each CLTL scenario.

**Figure 6:**
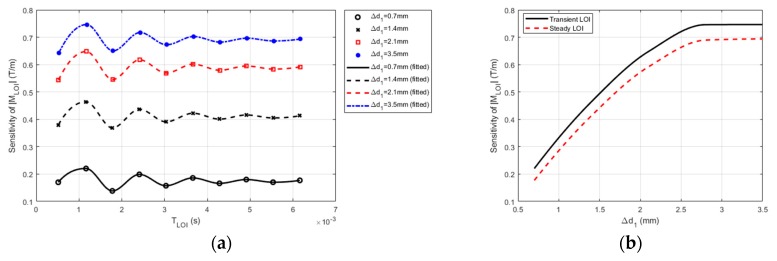
Sensitivities of the magnitudes of LOIs of PMEC signals vs. CLTL depth: (**a**) the sensitivity of M_LOI_ vs. T_LOI_ against ∆*d*_1_; (**b**) M_LOI_ of the transient and steady LOIs against ∆*d*_1._

It is noticeable from Figure 5b that the magnitude of the LOI point, |M_LOI_| in the function of T_LOI_ has significant transient characteristics in the presence of the cladded conductor. Consistent with the analysis results from Figure 5a, higher fluctuation in |M_LOI_| can be seen in early cycles of the carrier wave, whilst the LOI magnitude comes to a steady state at the late stage of the excitation when the time-harmonic features of the resulting PMEC signal *B_z_*, which are usually found in the signal of the traditional EC, can be observed. The dependency of |M_LOI_| on ∆*d*_1_ is also noticed in Figure 5b, which implies the potential application of |M_LOI_| for quantitative evaluation of the depth of CLTL, regardless of the variation in the probe LO. Because multiple LOI points can be extracted in PMEC signals whilst |M_LOI_| of one particular LOI is sufficiently applicable for CLTL evaluation, the unique LOI is sought based on the analysis in regard to the LOI sensitivity to ∆*d*_1_ (i.e., ∆|M_LOI_|/∆*d*_1_). The sensitivity analysis results are presented in Figure 6a.

It can be seen in Figure 6a that the sensitivity of the LOI magnitude varies with ∆*d*_1_. It is directly proportional to ∆*d*_1_, which is because of the enhancement of perturbation of eddy currents in the conductor in the presence of CLTL with its depth increased. For each CLTL case, fluctuation of the M_LOI_ sensitivity can also be observed in Figure 6a, which indicates that the optimal LOI point can be readily identified by finding the point with the maximum M_LOI_ sensitivity. For every CLTL scenario, the second LOI point is preferred for CLTL evaluation because of its highest sensitivity of M_LOI_ to the CLTL depth. Note that this optimal point is located in the transient section of the PMEC signal (0 ms ≤ *t* ≤ 5ms) and is thus called “transient LOI”. In contrast, when the PMEC signal reaches the pseudo time-harmonic state (5 ms ≤*t* ≤ 6.3 ms), the resulting LOI point extracted from the signal section is named “steady LOI”. The sensitivity of the magnitudes of the transient and steady LOIs to the CLTL depth is exhibited in Figure 6b. It can be observed from Figure 6b that the transient LOI has higher sensitivity to CLTL than the steady LOI. This indicates the advantage of LOI in PMEC signals over that of EC in assessment of hidden defects and is supportive of the findings regarding the PMEC superiority to EC in terms of the wide bandwidth of the field excitation and abundant information extracted from the signals for dedicated defect evaluation due to its transient features in testing signals.

Based on the investigation of characteristics of LOI in PMEC signals, the simultaneous evaluation for estimation of the CLTL depth and thickness of the protective coating is carried out in experiments. In light of the fact that M_LOI_ of the transient LOI is invariant with the probe LO and thus the protection-coating thickness is barely assessed, the peak value (PV) of the PMEC difference signal derived from subtraction of the signal for the defect-free specimen (i.e., the reference signal) from that with the thickness loss (i.e., the defect signal) is employed in inversion.

## 4. Experiments

**Figure 7:**
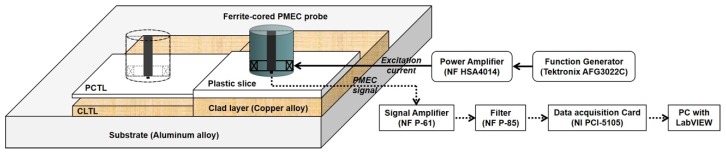
Schematic illustration of the PMEC system.

**Figure 8:**
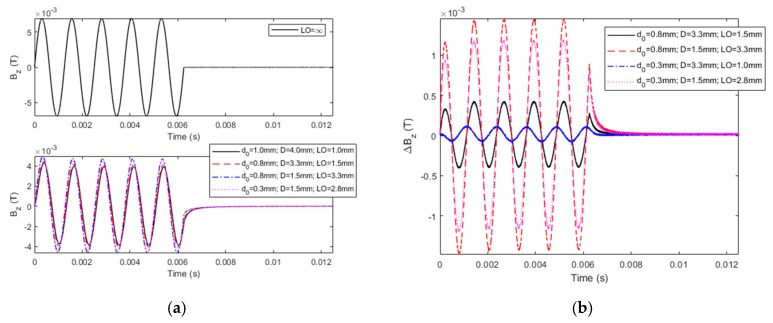
Experimental PMEC signals: (**a**) acquired signals of *B_z_*; (**b**) difference signals.

A PMEC system has been built for experimental investigation in regard to simultaneous assessment of the depths of CLTL and the protection-coating thickness loss (PCTL) in the featured cladded conductor. The schematic illustration of the system is presented in Figure 7. The parameters of the PMEC probe and defect-free specimen are the same as those listed in Table 1 and Table 2. The magnetic field sensor in the probe is the Hall device SS495A from Honeywell. The maximum amplitude of the excitation current, the carrier-wave frequency, and the pulse width and frequency of the modulation waves are 253 mA, 800 Hz, 6.3 ms, and 80 Hz, respectively. In an effort to simulate CLTL, the thickness of a clad layer of copper alloy varies from 0 to 3 mm, whilst a plastic slice with the thickness changing from 0 to 0.7 mm is adopted to simulate PCTL. The acquired PMEC signals corresponding to different depths of CLTL and PCTL, and the signal without the specimen (LO = ∞) are shown in Figure 8a, whilst the difference signals are portrayed in Figure 8b. After the difference signals are obtained, PVs are extracted. It is noted that each PV is corrected by multiplying its original value with the coefficient derived from PV_sim_/PV_exp_, where PV_sim_ and PV_exp_ denote the predicted and experimental peak values of the difference signals by subtracting the defect-free signal (∆*d*_0_ = ∆*d*_1_ = 0 mm) from the signal for LO = ∞, respectively.

**Figure 9:**
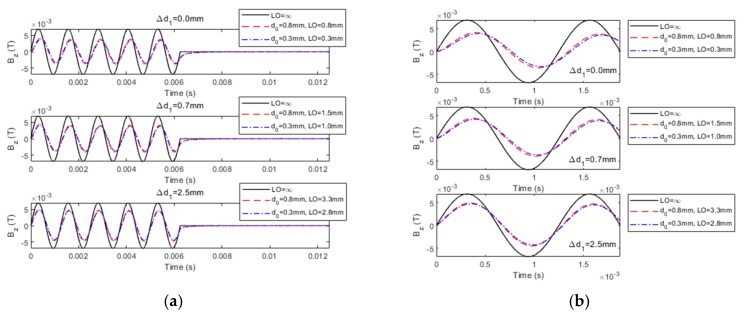
Testing signals indicating LOIs vs. ∆*d*_1_ against differential LOs: (**a**) signals in the one-cycle excitation; (**b**) zoom-in curves in the temporal window of 0 ms ≤ *t* ≤ 2 ms.

In a bid to localize the LOI point in the testing signal, the probe is first put in the air for acquisition of the air signal, which corresponds to the case of infinite LO. Following this, the PMEC signal is obtained with the probe deployed above the specimen and the intersection points of the signal with the air signal are extracted. LOIs of the testing signals for every CLTL are shown in Figure 9. For each scenario, |M_LOI_| of the transient LOI is acquired and regarded as the observed value for inversion. It is noted that correction of the experimental |M_LOI_| is also conducted. The raw |M_LOI_| is multiplied by the coefficient of |M_LOI_|_sim_/|M_LOI_|_exp_, where |M_LOI_|_sim_ and |M_LOI_|_exp_ denote the predicted and experimental magnitudes of the transient LOI between the defect-free and air signals, respectively.

**Figure 10:**
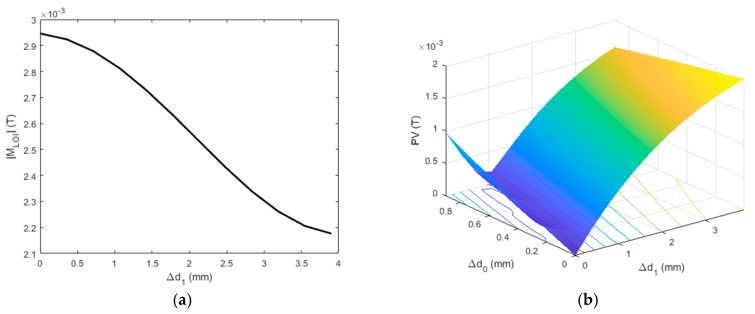
The established databases via the forward model: (**a**) the calibration curve of |M_LOI_| = *f*(∆*d*_1_); (**b**) the database of peak value (PV) = *f*(∆*d*_0_, ∆*d*_1_).

The corrections of measured PV and |M_LOI_| make the forward model, and in particular Equation (13), applicable for assessment of ∆*d*_0_ and ∆*d*_1_ regarding PCTL and CLTL, respectively. Thanks to the intrinsic characteristics of LOI, ∆*d*_0_ and ∆*d*_1_ can be decoupled, and thus the inverse process for approximation of ∆*d*_0_ and ∆*d*_1_ becomes straightforward. Based on Equation (13), the monotonic correlation of |M_LOI_| with ∆*d*_1_ can be established and presented in Figure 10a, which is independent of ∆*d*_0_ and can be formulated as |M_LOI_| = *f*(∆*d*_1_). Therefore, by using the observed |M_LOI_|, the thickness loss in the clad layer can be estimated by finding the root of *f*(∆*d*_1_^est^) = |M_LOI_|^obs^, where ∆*d*_1_^est^ and |M_LOI_|^obs^ are the approximated depth of CLTL and observed magnitude of the transient LOI, respectively. After ∆*d*_1_ is inversely retrieved, ∆*d*_0_ can subsequently be estimated by “looking it up” in the database, which is built up using Equation (13) and depicts the correlation between PV and the combination of (∆*d*_0_, ∆*d*_1_). The established database is presented in Figure 10b. The solution to ∆*d*_0_ can be efficiently sought by using the database along with ∆*d*_1_^est^, which gives the subspace of PV = *f*(∆*d*_0_, ∆*d*_1_^est^). The depth of PCTL can thus be evaluated by finding the root of *f*(∆*d*_0_^est^, ∆*d*_1_^est^) = PV^obs^, where PV^obs^ denotes the observed PV from experiments. With respect to each thick-loss case, |M_LOI_|^obs^, PV^obs^, and the estimated depths of CLTL and PCTL are tabulated in Table 3 along with the true values.

It can be observed from Table 3 that the estimated ∆*d*_0_ and ∆*d*_1_ agree well with the corresponding true values. Further analysis reveals that the evaluation accuracy regarding the thickness-loss cases is more than 94%, whilst the maximum relative error is found for ∆*d*_0_^est^ of Case #5, which is 5.7%. It is believed that the discrepancy between the approximated and true values results mostly from: (1) the extraneous noise in experiments; and (2) the small gap between the probe and protective coating during inspection, which is barely taken into account in the forward modeling. It is also noteworthy that the deviation of the measured conductivities of the clad layer and substrate (listed in Table 2 and used in the forward modeling) against the apparent conductivities at the probe position could undermine the evaluation accuracy. Based on the current investigation, it is suggested that for high-accuracy evaluation of CLTL and PCTL, the precision of the conductivity measurement regarding the reference materials of the clad layer and substrate be over 0.1 MS/m. Furthermore, it can be seen from Table 3 that the relative error of ∆*d*_0_^est^ is slightly higher than that of ∆*d*_1_^est^. This is because |M_LOI_| of the acquired transient LOI is immune to the variation in the probe LO, which is inevitable in PMEC inspection. In contrast, even though correction of PV is exploited, the LO variation brings about a small deviation of measured PV from the predicted value. In addition, the relative error of ∆*d*_0_^est^ is also accumulated from that of ∆*d*_1_^est^. Nonetheless, it is noticeable from the theoretical and experimental investigations that the LOI point in PMEC signals benefits the dedicated evaluation of hidden thickness loss in the cladded conductor in the virtue of the LO-invariant characteristics of LOI. In conjunction with PV of the PMEC difference signal, the simultaneous evaluation of depths of CLTL and PCTL, particularly in the case studies, is realized via efficient inversion based on the magnitude of the transient LOI.

It should be pointed out, that the proposed evaluation method for simultaneous assessment of depths of CLTL and PCTL is applicable for the cladded conductors, which are planar structures in lieu of tubular structures. It can barely be utilized for evaluation of the thickness loss taking place at the back surface of the cladded conductor (particularly the substrate), since the eddy current can hardly penetrate into the substrate due to the “shielding effect” of the clad layer, particularly with higher conductivity. In such case, the evaluation of the thickness loss is almost formidable because of considerably low sensitivity of the eddy current as well as the testing signal to the thickness loss in the back surface of the substrate. An alternative evaluation method should be applied in conjunction with the intensive investigation regarding the conductivity ratio i.e., *σ*_1_/*σ*_2_. In addition, it is noteworthy that the proposed method is inapplicable for the cladded conductors with considerable larger protection-coating thickness (in the order of centimeters). This is because for the case with the thick coating thickness, the incident magnetic field over the surface of the clad layer is too feeble to induce eddy currents for interrogation of the thickness loss in the conductive media involving the clad layer and substrate.

## 5. Conclusions

In this paper, the characteristics of LOI in signal responses from the ferrite-cored PMEC probe to the cladded conductor with its clad layer and protective coating prone to thickness loss has been intensively investigated. The closed-form expression of the PMEC signal from the magnetic field sensor of the probe has been formulated via ETREE modeling. Based on this, through a series of simulations, the amplitude and time instant of each LOI point in the PMEC signal in the presence of CLTL and PCTL have been analyzed. It has been found that the LOI point of PMEC has the transient feature at the early cycles of the excitation, whilst its time-harmonic characteristic is observed when the excitation comes to the steady state. Through sensitivity analysis, compared with the steady LOI, the transient LOI is identified as the optimal one and is preferred for assessment of depth of CLTL regardless of the variation in the probe LO. Following the theoretical investigation, experiments have been carried out with the PMEC system. In a bid to simultaneously evaluate the thickness loss in the clad layer and protective coating of the featured cladded conductor, the magnitude of the transient LOI was utilized along with PV of the PMEC difference signal. It is noticeable that, thanks to LOI characteristics, the influences of depths of CLTL and PCTL on the testing signals can be decoupled, which consequently makes the inversion for approximation of ∆*d*_0_ and ∆*d*_1_ straightforward. The case studies in experiments reveal that the efficient inverse scheme based on |M_LOI_| and PV is applicable for quantitative evaluation regarding the thickness loss in cladded conductors without much loss in accuracy.

## Figures and Tables

**Table 1:** Parameters of the PMEC probe.

Parameter	Value
Inner radius of the excitation coil, *r*_1_ (mm)	9.3
Outer radius of the excitation coil, *r*_2_ (mm)	18.4
Height of the excitation coil, *H* (mm)	8.8
Number of turns of the excitation coil, *N*	408
Radius of the ferrite core, *c* (mm)	4.9
Height of the ferrite core, *L* (mm)	12.7
Relative permeability of the ferrite core, *μ_c_*	142
Distance between the ferrite-core bottom and upper surface of the sensing element, *c*_1_ (mm)	0.9
Thickness of the sensing element *c*_2_-*c*_1_ (mm)	0.4
Radius of the sensing element, *r*_0_ (mm)	0.3

**Table 2:** Parameters of the specimen.

Parameter	Value
Conductivity of the clad layer, *σ*_1_ (MS/m)	19.8 *
Thickness of the clad layer, *d*_2_-*d*_1_ (mm)	4.0
Conductivity of the substrate, *σ*_2_ (MS/m)	34.4 *
Thickness of the substrate, *d*_3_-*d*_2_ (mm)	10.0
Thickness of the protective coating, *d*_0_ (mm)	1.0
Side length of the specimen, 2*h* (mm)	350.0

* the averaged value from multiple measured conductivities at various positions of the reference sample via direct current potential drop method.

**Table 3:** Comparison of ∆*d*_0_^est^ and ∆*d*_1_^est^ with the true values.

	Case #1	Case #2	Case #3	Case #4	Case #5
Observed values [|M_LOI_|^obs^, PV^obs^]/G	[29.46, 7.51]	[28.78, 4.17]	[28.77, 0.97]	[24.31, 14.83]	[24.32, 12.02]
Estimated thickness-loss depths [∆*d*_0_^est^, ∆*d*_1_^est^]/mm	[0.69, 0.01]	[0.21, 0.73]	[0.73, 0.72]	[0.21, 2.45]	[0.74, 2.53]
True values [∆*d*_0_, ∆*d*_1_]/mm	[0.7, 0.0]	[0.2, 0.7]	[0.7, 0.7]	[0.2, 2.5]	[0.7, 2.5]
